# Potential physiological tolerance mechanisms in faba bean to *Orobanche* spp. parasitism

**DOI:** 10.3389/fpls.2024.1497303

**Published:** 2024-12-06

**Authors:** Siwar Thebti, Amal Bouallegue, Touhami Rzigui, Youness En-Nahli, Faouzi Horchani, Taoufik Hosni, Mohamed Kharrat, Moez Amri, Zouhaier Abbes

**Affiliations:** ^1^ Field Crops Laboratory, National Institute for Agricultural Research of Tunisia (INRAT), Carthage University, Ariana, Tunisia; ^2^ Faculty of Sciences of Tunis, University of Tunis-El Manar, Tunis, Tunisia; ^3^ National Research Institute for Rural Engineering, Waters and Forestry (INRGREF), Ariana, Tunisia; ^4^ AgroBioSciences Program (AgBS), College of Agriculture and Environmental Science (CAES), University Mohammed VI Polytechnic (UM6P), Ben Guerir, Morocco; ^5^ Laboratory of Biotechnology and Biomonitoring of the Environment and Oasis Ecosystems, Faculty of Sciences of Gafsa, University of Gafsa, Gafsa, Tunisia

**Keywords:** *Vicia faba* L., *Orobanche crenata*, *Orobanche foetida*, tolerance, Fv/Fm, net carbon assimilation (An), transpiration (E)

## Abstract

*Orobanche* spp. are root parasitic plants that cause severe yield losses in faba bean (*Vicia faba* L.). The use of tolerant varieties remains a pivotal component of a successful integrated control strategy. In this study, we investigated the potential physiological mechanisms associated with tolerance to *O. crenata* and *O. foetida* in faba bean. The results showed that *Orobanche* parasitism significantly affected faba bean plants’ growth and seed production, especially in the sensitive Bachaar variety (up to 61.77% and 83.53% in shoot dry weight, up to 79.59% in pod number and no pod development when infected with *O. foetida* and *O. crenata*, respectively). This reduction was correlated with photosynthetic capacity (A_max_) decreases in response to both *O. foetida* and *O. crenata* parasitism. This decrease was highly pronounced in the sensitive Bachaar variety with 24.57% and 63.43% decreases, respectively. Significant decreases were also observed in the sensitive Bachaar cultivar for the photochemical efficiency of PSII (F_v_/F_m_) (1.1% and 4.78%), the maximum transpiration (E_max_) (11.8% and 39.13%), and the maximum water use efficiency (WUE_max_) (24.97% and 41.77%) in response to *O. foetida* and *O. crenata* parasitism, respectively, compared to non-significant differences for the tolerant Chams, Chourouk, and Zaher varieties. The tolerant faba bean varieties were able to maintain a normal function of their photosynthesis capacity (A_n_) and conserve their growth and seed production level as a result of an acclimation to parasitic attack (Maintaining WUE_max_). Our results suggest that yield components such as shoot dry weight, pod and leaf numbers, and photosynthetic parameters, notably the transpiration rate, can serve as suitable traits for assessing tolerance to *Orobanche* parasitism in faba bean plants.

## 1 Introduction

Legume production in the Mediterranean countries is significantly affected by broomrape infection which can lead to substantial yield losses under high infection levels. In Tunisia, the faba bean is the most cultivated legume with a total production of 65 thousand tons and a cultivated area of 48 thousand hectares, representing 75% of the total grown grain legume area (DGPA, 2022). Depending on the infection level, the genotype, and environmental conditions, yield losses caused by *Orobanche* infection can reach 90% (Trabelsi et al., 2015; Bouraoui et al., 2016) or even 100% (complete loss) (Amri et al., 2021). Over 50,000 hectares were reported to be infected by different broomrape species in Tunisia (Amri et al., 2019). *O. crenata* and *O. foetida* are the two major species of *Orobanche* that cause important damage and limit the development of various legume crops, resulting in a significant decline in food legume production and productivity (Amri et al., 2019, 2021).

Several control methods have been tested, including chemical and biological methods, late sowing, trap crops, intercropping, and manual uprooting (Bouraoui et al., 2016; Abbes et al., 2010a, 2019). However, most of these methods have proven to be partly effective (Amri et al., 2019; Abbes et al., 2019). The development of tolerant varieties remains the most effective control method for parasitic weed management (Triki et al., 2018; Amri et al., 2019; Abbes et al., 2019). Over the last three decades, research efforts into *Orobanche* tolerance have intensified in Tunisia, resulting in the development and release of four tolerant faba bean varieties, i.e., Najeh, Chourouk, Chams, and Zaher, which have tolerance for both *O. crenata* and *O. foetida* (Abbes et al., 2007; Kharrat et al., 2010; Amri et al., 2019). These varieties showed low *Orobanche* seed germination stimulant production, a limited attachment number of germinated seeds, and reduced growth of established tubercles, resulting in a low number of emerged shoots of *Orobanche* in the host plant (Abbes et al., 2009a, b, 2010b, 2020; Trabelsi et al., 2016, 2017).

In order to evaluate the impact of parasitic weeds on their hosts at earlier development stages, several yield components and physiological parameters were studied. Rousseau et al. (2015) found that plant infection by root parasitic weeds has a systemic impact that can be observed on the host’s leaves. Previous investigations demonstrated that *Orobanche* parasitism affected the host leaves’ chlorophyll concentrations and photosynthetic activities such as the quantum efficiency of PSII (Mauromicale et al., 2008; Vrbničanin et al., 2013; Nefzi et al., 2016; Abbes et al., 2020; Amri et al., 2021). Studies of hemiparasitic associations, such as *Striga*, *Rhinanthus*, and *Cassytha*, found lower soluble protein concentrations (Rubisco content) and lower chlorophyll concentrations that might be responsible for decreased host photosynthesis (Watling and Press, 2000; Cameron et al., 2005; Shen et al., 2010). Shen et al. (2007) found that *Cuscuta campestris* can affect *Mikania micrantha* photosynthesis through both an adverse impact on stomatal conductance (gs) and direct effects on photosynthetic metabolism, such as carboxylation efficiency and CO_2_-saturated rate of photosynthesis. Amri et al. (2021) observed a significant positive correlation between photosynthetic parameters [the chlorophyll content index and the maximum quantum efficiency (F_v_/F_m_)] and tolerance to *O. crenata* and *O. foetida* in faba bean. The same authors suggested considering these physiological traits in plant breeding and screening for tolerance to broomrapes. Few studies have focused on the effects of *Orobanche* on parameters related to the photosynthetic capacity of faba bean leaves (Amri et al., 2021; Ennami et al., 2020). In the present study, we examined the physiological mechanisms associated with tolerance to both *O. crenata* and *O. foetida* in three tolerant and one sensitive faba bean varieties and elucidated the potential correlations between *Orobanche* tolerance and photosynthetic activities in faba bean.

## 2 Materials and methods

### 2.1 Plant material

Four faba bean (*Vicia faba* L.) varieties were used in this study; three varieties, namely, Chourouk, Chams, and Zaher, are known for their tolerance to *Ascochyta*, *Botrytis*, rust, and both *O. foetida* and *O. crenata* (Kharrat et al., 2010; Amri et al., 2019), while the variety Bachaar is known for its sensitivity to both *Orobanche* species. All these varieties displayed high productivity in *Orobanche*-free soils. All faba bean seeds were provided by the Field Crops Laboratory, National Institute of Agricultural Research (INRAT), Tunisia. *O. foetida* and *O. crenata* seeds were collected in Tunisia from mature shoots in faba bean fields in the Beja (Oued Beja Agricultural Experimental Unit in north-west Tunisia) and Ariana regions, respectively.

### 2.2 Pot experiments

Pot experiments were carried out to evaluate the response of the three tolerant faba bean varieties, Chourouk, Chams, and Zaher, to *O. foetida* and *O. crenata* parasitism. Seeds of the different faba bean varieties were surface sterilized with calcium hypochlorite (1%) for 15 minutes and then rinsed four times with sterilized distilled water. Artificial inoculation was performed by uniformly mixing 25 mg of *O. foetida* or *O. crenata* per 1 kg of soil in 2 L capacity pots (approximately 12,500 *Orobanche* seeds per pot). Pots containing *Orobanche*-free soil were used as control. Two faba bean seeds were sown in each pot and reduced to only seedlings after emergence (1 week after sowing). Five replications/pots per variety were used for the inoculated and non-inoculated pots. The experiment was conducted under greenhouse conditions at 20 ± 3°C with a humidity of 70% and a 16/8 h photoperiod. The faba bean plants were irrigated regularly with tap water to maintain soil moisture.

Photosynthetic active radiation was measured on six node leaves using a Li-190 Quantum Sensor (Li-Cor Bioscience, Lincoln, NE, USA) with a 330-360 μmol/(m^2^s) range.

### 2.3 Data collection

#### 2.3.1 Faba bean yield components and *Orobanche* infection

At the pod setting stage (4 months after planting), the faba bean plants were uprooted and washed carefully. The total attachment number (TON) was counted and classified according to their development stage into underground/non-emerged (S1-S4) (NEO) or emerged *Orobanche* tubercles/shoots (S5) (EON). S1, S2, S3, S4, and S5 correspond to attachment of the haustorium to the host root, small tubercles without root formation, tubercles on the crown root without shoot development, shoot formation remaining underground, and emergence, respectively (Abbes et al., 2011). In addition, faba bean leaf (LN/P) and pod numbers (PN/P) and shoot height (SH) were determined. The leaves were directly weighed to obtain the fresh mass (MF), and then incubated in distilled water for 24h to obtain the turgid mass (MT) before being dried in an oven at 80°C for 72h to measure the dry mass (MD). The relative water content (RWC) was calculated using the following equation:


RWC=((MF/MD)/(MT/MD))∗100 

(Barrs and Weatherley, 1962)

The root (RDW/P), shoot (ShDW/P), stem (SDW/P), leaf (LDW/P), pod (PDW/P), and *Orobanche* dry weights (ODW/P) per plant (g) were recorded after drying in an oven at 80°C for 72h.

#### 2.3.2 Chlorophyll fluorescence

*In vivo*, chlorophyll *a* fluorescence emissions in 30-min dark-adapted leaves (the 6^th^ well-developed node leaf) were measured with a portable modulated chlorophyll fluorometer (Opti-Sciences, OS-30p+, Malaysia) at the pod setting development stage (4 months after planting). After adaptation to the dark, the modulated fluorometer allows the accurate measurement of the minimum fluorescence (F_o_) and the maximum fluorescence (F_m_) using a weak, modulated light and a subsequent saturating flash of white light, respectively. The maximum photochemical efficiency of PSII was calculated as the ratio of the light-induced variable and the maximum fluorescence of chlorophyll: F_v_/F_m_= (F_m_-F_0_)/F_m_ (Lanquar et al., 2010).

#### 2.3.3 Photosynthetic gas exchange

The photosynthetic response to light levels was measured using Li-Cor 6400-40 equipped with a red-blue LED source (Li-Cor Inc., United States) at the pod setting development stage. All the measurements were carried out at the ambient CO_2_ concentration (400 ppm) and at 25°C on the 6^th^ well-developed node leaf for each plant. The vapor pressure deficit and air flow rate were kept at 1.2 ± 0.2 kPa and 300 cm^3^/min, respectively.

An incident light level of 600 μmol/(m^2^ s) PAR was used to reach a steady state in each leaf. Net CO_2_ assimilation (A_n_) was recorded at various levels of photosynthetic photon flux density (PPFD) once it became stable. Simultaneously, transpiration (E) was also recorded. Instantaneous water use efficiency (WUE) was calculated as A_n_/E (μmol CO_2_/μmol H_2_O). For the A_n_/PPFD curves, the light-saturated net photosynthesis rate (A_max_) was estimated from curves as the maximum photosynthetic rate (photosynthetic capacity), the apparent quantum yield (Φ) was calculated on the basis of incident light as the initial slope at the 3 lowest PPFD values, and the light compensation point (LCP) was estimated from the x-axis intercepts. Finally, specific leaf area (SLA) was determined as the ratio of leaf area to leaf dry mass of individual leaves.

### 2.4 Statistical analysis

The statistical analyses were conducted using R and SPSS software. Analysis of variance (ANOVA) was performed for all the studied traits, employing a general linear model with genotypes and treatment considered as fixed factors. Descriptive statistics, including the number of observations (n), mean, standard error (SE), and Duncan’s test were carried out using SPSS software.

R studio was utilized to generate the principal component analysis (PCA), dendogram, and Pearson’s correlation using the “factoextra”, “factoMineR”, and “metan” packages. PCA was conducted using data derived from stressed faba bean plants and non-stressed plants as controls. Pearson’s correlation coefficient was employed to examine the relationships between physiological and yield component variables. All measurements were carried out in triplicate, significance levels were set at P = 0.05, and Duncan’s multiple-range test was employed for pairwise comparisons.

## 3 Results

### 3.1 Effect of *Orobanche* parasitism on host plant growth

Significant differences were observed between the sensitive and tolerant varieties in response to both *O. crenata* and *O. foetida* parasitism. The results demonstrated significant differences (P ≤ 0.05) among the tested varieties for TON, ODW, NEO, SDW, LN, SLA, and RWC, while no significant variations were observed for the other traits. The treatments exhibited a significant influence on all the yield components except SLA. The interaction between genotype and the treatment was statistically significant for TON, ODW, NEO, RDW, ShDW, SDW, LDW, LN, PN, and SLA (**Table 1**).

**Table 1:** *Orobanche* infection parameters and yield components of faba bean varieties.

	TON	ODW	NEO	EON	RDW	ShDW	SDW	LDW	PDW	LN	PN	SH	SLA	RWC
Genotype (G)	12.25***	5.99**	12.63***	1.63ns	0.79ns	0.77ns	4.94**	0.57ns	1.84ns	4.14*	0.44ns	0.85ns	25.15***	9.06***
Treatment (T)	56.44***	35.99***	46.01***	16.68***	10.10***	44.96***	51***	16.86***	23.94***	17.6***	55.31***	0.90***	0.75ns	7.68**
G*T	5.15***	3.17*	5.13***	0.92ns	3.27**	4.7***	5.14***	4.73***	1.75ns	8.14***	5.12***	1.53ns	4.48**	2.28ns

***, significant at the 0.001 level; **, significant at the 0.01 level; *, significant at the 0.05 level; ns, not significant (Tukey’s test).

Total *Orobanche* tubercle number, TON (S1-S5); *Orobanche* dry weight, ODW; non-emerged (underground) *Orobanche* tubercles, NEO; Emerged *Orobanche* number, EON; root dry weight, RDW; shoot dry weight, ShDW; stem dry weight, SDW; leaf dry weight, LDW; pod dry weight, PDW; Leaf number, LN; Pod number, PN; Shoot height, SH; specific leaf area, SLA; relative water content, RWC.

**Figure 1:**
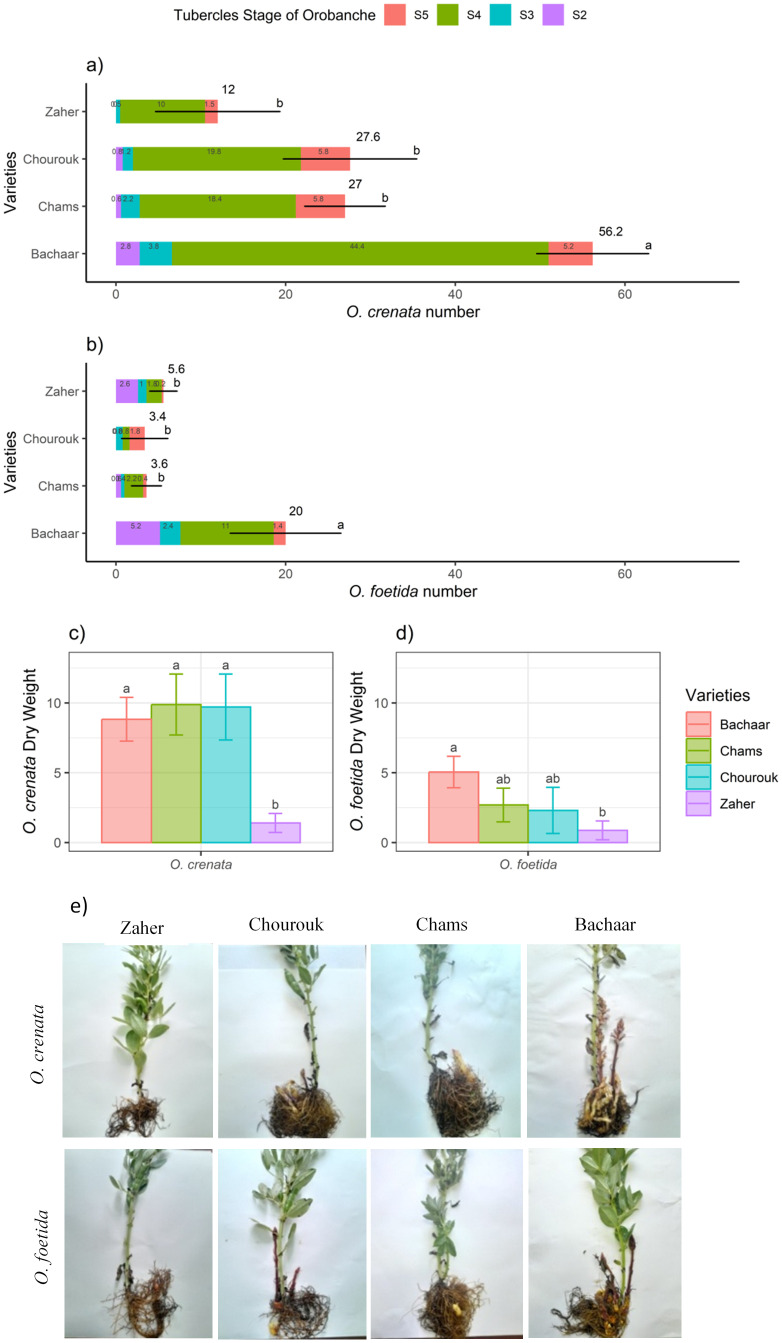
The total attachment number and attachment number for each stage **(A, B)**, the dry weight **(C, D)**, and pictures **(E)** of *O. crenata* and *O. foetida* infection in the tolerant (Chourouk, Chams, and Zaher) and sensitive (Bachaar) faba bean (*Vicia faba* L.) varieties in pots. Values are means ± SE of at least three independent measurements. Measurements were carried out 4 months after sowing.

The three tolerant varieties, Chourouk, Chams, and Zaher, showed lower *O. foetida* and *O. crenata* attachments (TON) compared to the sensitive Bachaar variety. The majority of these attachments were non-emerged tubercles (NEO). There were significant differences in the number of attachments progressing from stage 2 to stage 4 and no significant differences in the number of attachments progressing to stage S5 between the tolerant varieties and the sensitive variety (Bachaar) (**Figures 1A, B**). The *Orobanche* DW did not exhibit statistically significant differences among the various varieties, except for Zaher, where the *O. crenata* and *O. foetida* DWs were significantly lower than for the Bachaar variety (**Figures 1C, D**). For all varieties, the TON, NEO, and ODW were significantly higher in the presence of *O. crenata* as compared to *O. foetida* (**Figure 1**). It is worth noting that no necrosis of the attachments/tubercles was observed during this experiment.

In the absence of infection, there were significant variations in RDW, LN, and PN per plant among the tolerant varieties Chourouk, Chams, and Zaher compared to the sensitive Bachaar variety. Among these varieties, Bachaar demonstrated the highest productivity with 19.6 pods per plant (**Table 2**).

**Table 2:** Yield components of tolerant (Chams, Chourouk, and Zaher) and sensitive (Bachaar) faba bean (*Vicia faba* L.) varieties infected or not by *O. foetida* and *O. crenata* in pots. For the abbreviations, see **Table 1**.

Parameter		RDW (g)	ShDW (g)	SDW (g)	LDW (g)	PDW (g)	LN	PN	SH	RWC	SAL
Variety	Treatment										
Bachaar	Control	8.93 ± 2.37^b^	19.44 ± 1.72^g^	7.53 ± 0.88^d^	6.82 ± 1.03^e^	5.08 ± 0.52^d^	35.2± 2.67^d^	19.6± 1.36^f^	85.6± 5.42^d^	62.91± 2.72^bd^	0.074 ± 0.017^de^
	*O. foetida*	2.65 ± 0.27^a^	7.43 ± 0.71^ad^	3.15 ± 0.38^b^	3.73 ± 0.16^bd^	1.82 ± 0.88^ab^	20.33± 0.66^bc^	4.00± 1.52^ac^	66.4± 2.3^ab^	51.86± 2.62^a^	0.078 ± 0.012^e^
	*O. crenata*	1.53 ± 0.72^a^	3.2 ± 1.44^a^	1.57 ± 0.5^a^	1.62 ± 0.94^a^	0.00± 0.00^a^	12.66± 3.66^a^	0.00± 0.00^a^	53± 8.08^a^	56.15± 3.78^ac^	0.124 ± 0.007^f^
Chams	Control	4.47 ± 0.43^a^	13.73 ± 2.2^ef^	4.78 ± 0.68^c^	3.88 ± 0.55^bd^	5.07 ± 1.08^d^	19± 0.7^bc^	10.8± 1.94^e^	69± 4.84^bc^	67.51± 2.85^de^	0.045 ± 0.008^ad^
	*O. foetida*	3.86 ± 0.8^a^	12.83 ± 0.88^ef^	3.64± 0.49^bc^	4.56 ± 0.75^cd^	4.63 ± 0.66^cd^	20± 0.09^bc^	7.8± 2.22^ce^	69.8± 6.33^bc^	60.95± 2.32^bd^	0.063± 0.017^ce^
	*O. crenata*	3.09 ± 1.46^a^	5.77 ± 1.56^ab^	2.19 ± 0.53^ab^	2.57 ± 0.29^ab^	1.008 ± 0.86^a^	17.4± 0.97^ab^	2.2± 1.74^ab^	54.4± 5.87^a^	72.28± 1.69^e^	0.038 ± 0.008^ac^
Chourouk	Control	4.47 ± 0.4^a^	16.42 ± 1.88^fg^	6.88 ± 0.43^d^	5.37 ± 0.69^de^	4.16± 0.9^bd^	24.8± 4.83^c^	12.2± 1.88^e^	81± 2.84^cd^	56.73± 1.24^ac^	0.0820 ± 0.01^e^
	*O. foetida*	4.26 ± 0.65^a^	10.5 ± 1.02^ce^	3.66 ± 0.34^bc^	2.67 ± 0.26^ab^	3.51 ± 0.71^bd^	20.84± 0.72^bc^	6.2± 1.24^bd^	68.4± 1.56^bc^	58.34± 3.16^ac^	0.047 ± 0.002^ad^
	*O. crenata*	2.96 ± 0.56^a^	7.27 ± 1.01^ac^	3.09 ± 0.35^b^	3.38 ± 0.47^ac^	0.806 ± 0.38^a^	19± 1.34^bc^	3.4± 1.4^ac^	60.4± 3.41^ab^	61.69± 2.82^bd^	0.0531 ± 0.005^be^
Zaher	Control	4.19 ± 0.78^a^	11.8 ± 1.85^de^	3.73 ± 0.54^bc^	3.72 ± 0.64^bd^	4.35 ± 0.67^cd^	18.02± 0.66^ab^	10.6± 1.02^de^	73.6± 5.86^bd^	56.47± 2.68^ac^	0.021 ± 0.0006^a^
	*O. foetida*	2.36 ± 0.16^a^	10.13 ± 1.24^be^	3.05 ± 0.31^b^	3.8 ± 0.6^bc^	2.69± 0.95^ac^	19.2± 0.48^bc^	5.2± 1.65^bc^	69± 3.2^bc^	55.45± 2.91^ab^	0.024 ± 0.00003^ab^
	*O. crenata*	3 ± 0.17^a^	7.65 ± 1.05^bd^	2.43 ± 0.22^ab^	2.87 ± 0.21^ac^	2.35 ± 0.79^ac^	17.25± 0.73^ab^	4.25± 0.73^ac^	61.5± 3.05^ab^	64.51± 1.44^cd^	0.030 ± 0.003^ab^

Data are means ± SE. Means with the same letters within a column are not significantly different at p = 0.05 (Duncan’s test).

Under *Orobanche* spp. infection, there was a clear decrease in all yield components for the sensitive Bachaar variety. The most substantial decrease was observed in ShDW, with decreases of 61.77% and 83.53% under *O. foetida* and *O. crenata* infections, respectively. Additionally, no pod development was observed for *O. crenata-*infected plants, while only four pods per plant were recorded under *O. foetida* infection. In contrast, the three tolerant varieties displayed greater resilience to infection by both *Orobanche* species. Notably, there was no significant reduction observed in RDW and LN in the tolerant varieties. The decrease in ShDW was limited to 36.05% and 55.72% for Chourouk, 6.55% and 57.97% for Chams, and 14.15% and 35.16% for Zaher when infected by *O. foetida* and *O. crenata*, respectively. Among these tolerant varieties, in comparison with the non-infected plants, no significant reduction in pod DW was observed in response to *O. foetida* infection. However, when parasitized by *O. crenata*, the pod number and DW were significantly affected except for Zaher, which showed no significant decrease in pod dry weight. On average, it was observed that faba bean yield components were highly affected by *O. crenata* compared to *O. foetida* across the tolerant and sensitive varieties (**Table 2**). For all varieties, except for Bachaar infected with *O. foetida*, *Orobanche* parasitism had no significant effect on the RWC. Regarding the SLA, no significant changes were observed between the non-infected and infected plants, with the exception of Chourouk infected by *O. foetida*, which displayed a notable reduction of 42.68%. Conversely, Bachaar plants infected by *O. crenata* exhibited a high SLA (an increase of 67.56%).

### 3.2 Effect of *Orobanche* parasitism on the host physiological activities

Significant differences were observed among the tested varieties for F_v_/F_m_, A_max_, Φ, LCP, and E_max_, while no significant variations were observed for the other traits. The treatments exhibited a significant influence on A_max_, Φ, LCP, and E_max_, while it did not significantly affect F_v_/F_m_ and WUE_max_. Notably, for all the physiological parameters, the interaction between the genotype and the treatment was statistically significantly different (**Table 3**).

**Table 3:** Physiological traits of the faba bean varieties.

	F_v_/F_m_	A_max_	Φ	LCP	E_max_	WUE_max_
Genotype G	3.29*	15.29***	8.94***	23.47***	7.28***	2.42ns
Treatment T	1.01ns	24.01***	15.39***	16.14***	7.4**	2.09ns
G*T	2.8*	21.34***	18.08***	8.66***	3.15*	3.12*

***, significant at the 0.001 level; **, significant at the 0.01 level; *, significant at the 0.05 level; ns, not significant (Tukey’s test).

Maximal photochemical efficiency of PSII, Fv/Fm; light-saturated photosynthesis, A_max_; apparent quantum yield, Φ; light compensation point, LCP; maximal transpiration rate, E_max_; maximal water use efficiency, WUE_max_.

#### 3.2.1 Chlorophyll fluorescence

The F_v_/F_m_ ratio remained consistently near 0.8 in both the non-infected and infected plants of the Chourouk, Chams, and Zaher varieties. Interestingly, the Chourouk plants, even when infected by both Orobanche species, showed a significant increase in the F_v_/F_m_ ratio, indicating that PSII activity was not affected by *Orobanche* infection. However, infection, particularly by *O. crenata*, significantly decreased the F_v_/F_m_ ratio in the sensitive Bachaar variety, indicating that *Orobanche* parasitism indeed induces photoinhibition of Photosystem II in this sensitive variety (**Table 4**).

**Table 4:** Physiological traits in the leaves of tolerant (Chams, Chourouk, and Zaher) and sensitive (Bachaar) faba bean varieties.

Parameter		F_v_/F_m_	A_max_ (μmol CO_2_ /(m^2^s))	Φ (μmol CO_2_/mol photon)	LCP<i> </ι>(μmol photons/(m^2^s))	E_max_ (mmol/(m^2^s))	WUE_max_ (μmol CO_2_/μmol H_2_O)
Variety	Treatment						
Bachaar	Control	0.815 ± 0.004^c^	10.01 ± 0.37^h^	16.19 ± 0.37^a^	10.66 ± 2.68^ab^	1.61 ± 0.17^cd^	11.13 ± 1.54^d^
	*O. foetida*	0.806 ± 0.003^bc^	7.55 ± 0.07^ce^	20.51 ± 0.23^cd^	25 ± 5.77^c^	1.42 ± 0.01^bc^	8.35 ± 0.49^ac^
	*O. crenata*	0.776 ± 0.017^ab^	3.66 ± 0.34^a^	22.47 ± 0.45^e^	6.66 ± 0.18^a^	0.98 ± 0.00^a^	6.48 ± 0.82^a^
Chams	Control	0.811 ± 0.004^bc^	8.62 ± 0.29^eg^	20.12 ± 0.41^bd^	30 ± 0.31^c^	1.54 ± 0.17^bd^	10.27 ± 1.44^cd^
	*O. foetida*	0.823 ± 0.015^c^	9.04 ± 0.14^gh^	15.55 ± 0.65^a^	56.33 ± 7.24^d^	1.55 ± 0.09^bd^	9.36 ± 0.55^bd^
	*O. crenata*	0.816 ± 0.021^c^	7.13 ± 0.22^c^	20.55 ± 0.14^cd^	23.66 ± 1.49^c^	1.37 ± 0.07^bc^	8.63 ± 0.62^ad^
Chourouk	Control	0.762 ± 0.01^a^	8.79 ± 0.63^fg^	16.38 ± 0.3^a^	12.33 ± 2.24^ab^	1.74 ± 0.15^cd^	8.10 ± 0.43^ac^
	*O. foetida*	0.807 ± 0.008^bc^	8.42 ± 0. 34^dg^	19.08 ± 1^bc^	25.66 ± 1.01^c^	1.88 ± 0.10^d^	7.21 ± 0.18^ab^
	*O. crenata*	0.816 ± 0.021^c^	8.85 ± 0.78^fg^	18.51 ± 0.32^b^	27.66 ± 1.85^c^	1.74 ± 0.06^cd^	8.19 ± 0.47^ac^
Zaher	Control	0.815 ± 0.004^c^	7.28 ± 0.15^cd^	21.21 ± 0.79^de^	26.33 ± 5.14^c^	1.88 ± 0.13^d^	7.02 ± 0.36^ab^
	*O. foetida*	0.815 ± 0.006^c^	6.04 ± 0.2^b^	19.05 ± 0.81^bc^	20.33 ± 1.42^bc^	1.19 ± 0.09^ab^	6.94 ± 0.06^ab^
	*O. crenata*	0.831 ± 0.002^c^	7.8 ± 0.16^cf^	20 ± 0.33^bd^	24.33 ± 1.01^c^	1.35 ± 0.21^bc^	9.43 ± 1.48^bd^

Data are means ± SE. Means with the same letters within a column are not significantly different at p = 0.05 (Duncan test).

For the abbreviations, see **Table 3**.

#### 3.2.2 Photosynthetic gas exchange

**Figure 2:**
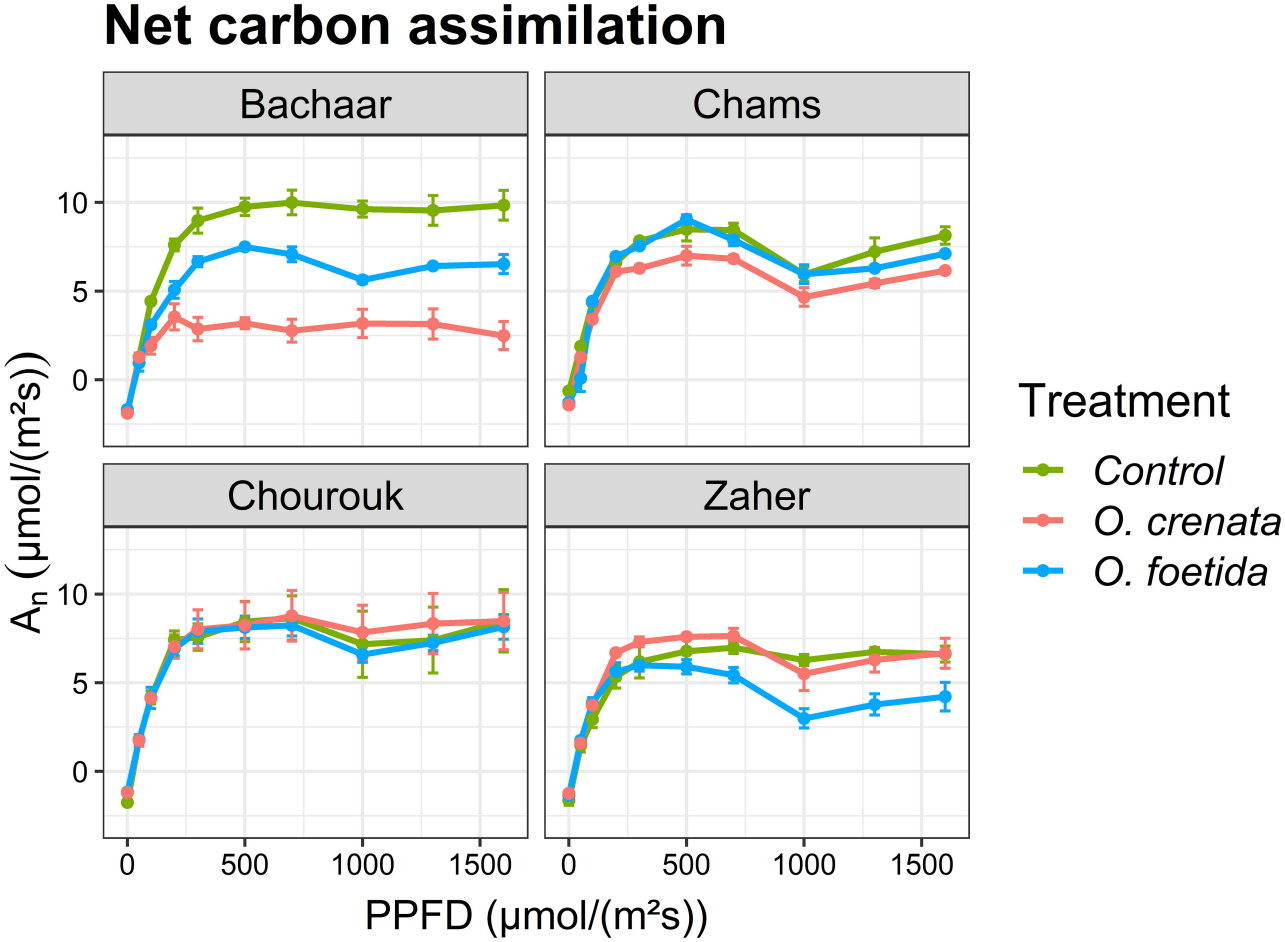
Light response curves: net carbon assimilation (A_n_) of attached leaves measured at different PPFD levels in an atmosphere of 400 ppm CO_2_ and 25°C for different faba bean varieties. Values are means ± SE of at least three independent measurements. Measurements were carried out 4 months after sowing.

**Figure 2** presents the light response curves of photosynthesis (net carbon assimilation A_n_) in the sixth fully expanded mature leaf of faba bean plants, with **Table 4** delineating specific photosynthetic parameters. The photosynthetic light curves exhibited a consistent trend among all treatments and varieties (**Figure 2**). A_n_ showed a proportionate increase in response to increasing PPFD until reaching a light saturation of 400 μmol/(m^2^s). Regarding the tolerant varieties, the A_n_ in the 6^th^ leaf exhibited no significant variation between the non-infected and infected plants for both broomrape species, except for Chams plants infected by *O. crenata* and Zaher plants infected by *O. foetida* where A_n_ was significantly reduced. In contrast, for the sensitive Bachaar variety, A_n_ was significantly lower in the leaves of the infected plants compared to the non-infected ones. Similarly, *O. crenata* induced a more pronounced decrease in A_n_ within this particular variety when compared to the impact of *O. foetida.*

No significant differences were observed in A_max_ between the non-infected and infected plants of tolerant faba bean varieties, with the exception of Chams infected by *O. crenata* and Zaher infected by *O. foetida* (**Table 4**). However, A_max_ showed significant decreases in response to both *O. foetida* (24.57%) and *O. crenata* (63.43%) in the sensitive Bachaar variety. The Φ exhibited a significant increase, showing increases of 26.68% and 38.78% in the sensitive Bachaar variety, against only 16.48% and 13% in Chourouk when infected by *O. foetida* and *O. crenata*, respectively. Significant decreases were also recorded for Chams and Zaher plants infected by *O. foetida*. The results also showed a significant increase in the LCP in Chams and Bachaar plants infected by *O. foetida*, as well as Chourouk plants concurrently infected by both *Orobanche* species. The maximum increase in the LCP was observed in the sensitive Bachaar variety plants infected with *O. foetida*, exhibiting a substantial increase of 2.34-fold compared to the control.

#### 3.2.3 Transpiration and intrinsic water use efficiency

**Figure 3:**
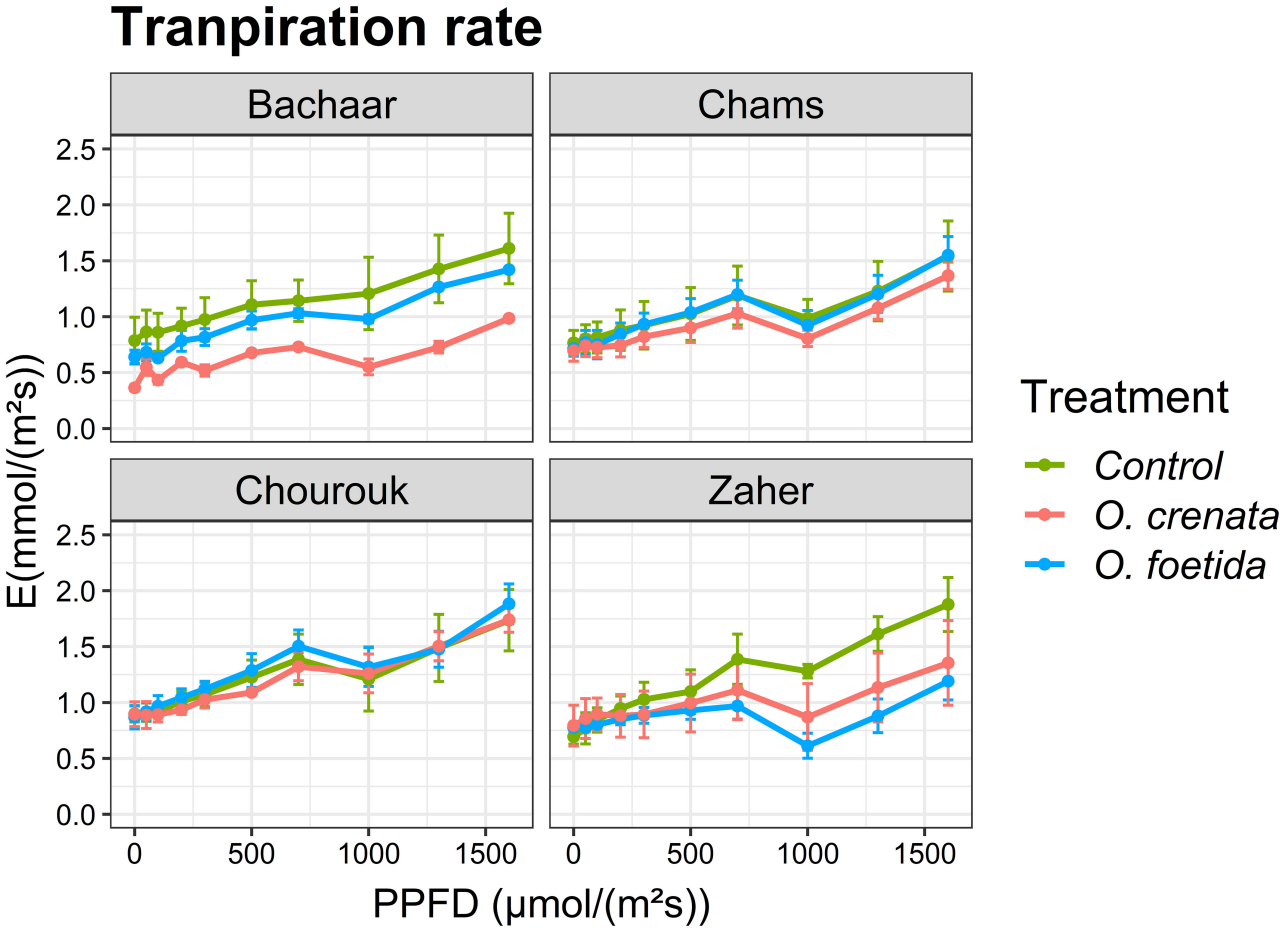
Response of gas exchange to light: transpiration rate (E) as a function of PPFD in an atmosphere of 400 ppm CO_2_ and 25°C for different faba bean varieties. Values are means ± SE of at least three independent measurements. Measurements were carried out 4 months after sowing.

**Figure 4:**
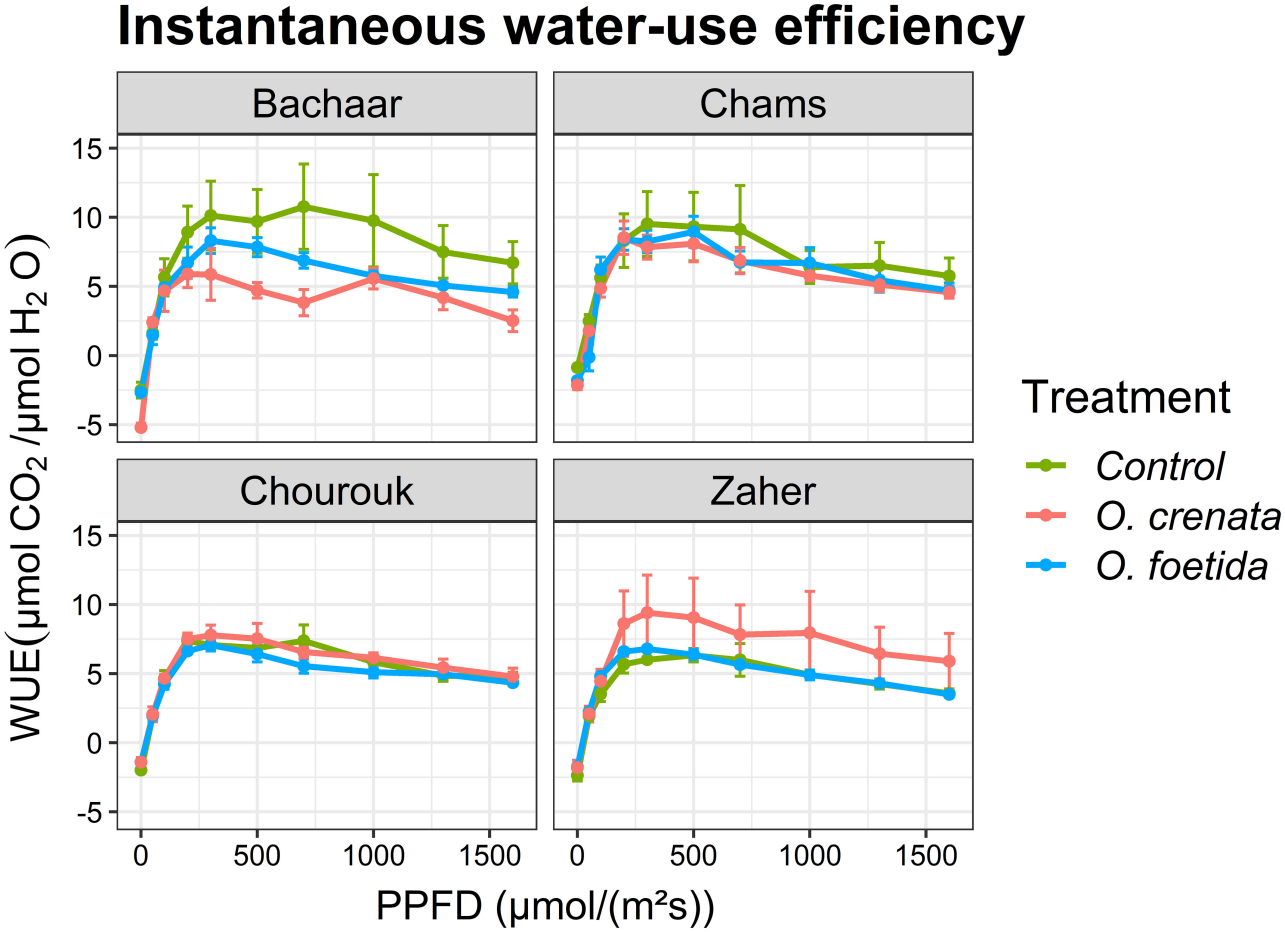
Response of gas exchange to light: instantaneous water-use efficiency (WUE) as a function of PPFD in an atmosphere of 400 ppm CO_2_ and 25°C for different faba bean varieties. Values are means ± SE of at least three independent measurements. Measurements were carried out 4 months after sowing.

The trends of E and intrinsic WUE responses to light were similar in tolerant and sensitive varieties (**Figures 3**, **4**, **Table 4**). Only the sensitive Bachaar and the tolerant Zaher varieties showed a significant decrease in E in response to *Orobanche* infection. However, in the case of intrinsic WUE, a significant decrease was observed exclusively in the sensitive Bachaar variety in response to infection with both *Orobanche* species.

### 3.3 Principal component analysis and correlations

In order to assess the performance of the four faba bean varieties under both non-infected and infected conditions, a comprehensive set of 20 yield components and physiological parameters was systematically analyzed. Subsequently, PCA and hierarchical cluster analysis (HCA) were performed to confirm the discrimination among faba bean varieties. Additionally, correlation analyses were conducted to investigate the relationships between the different variables.

**Figure 5:**
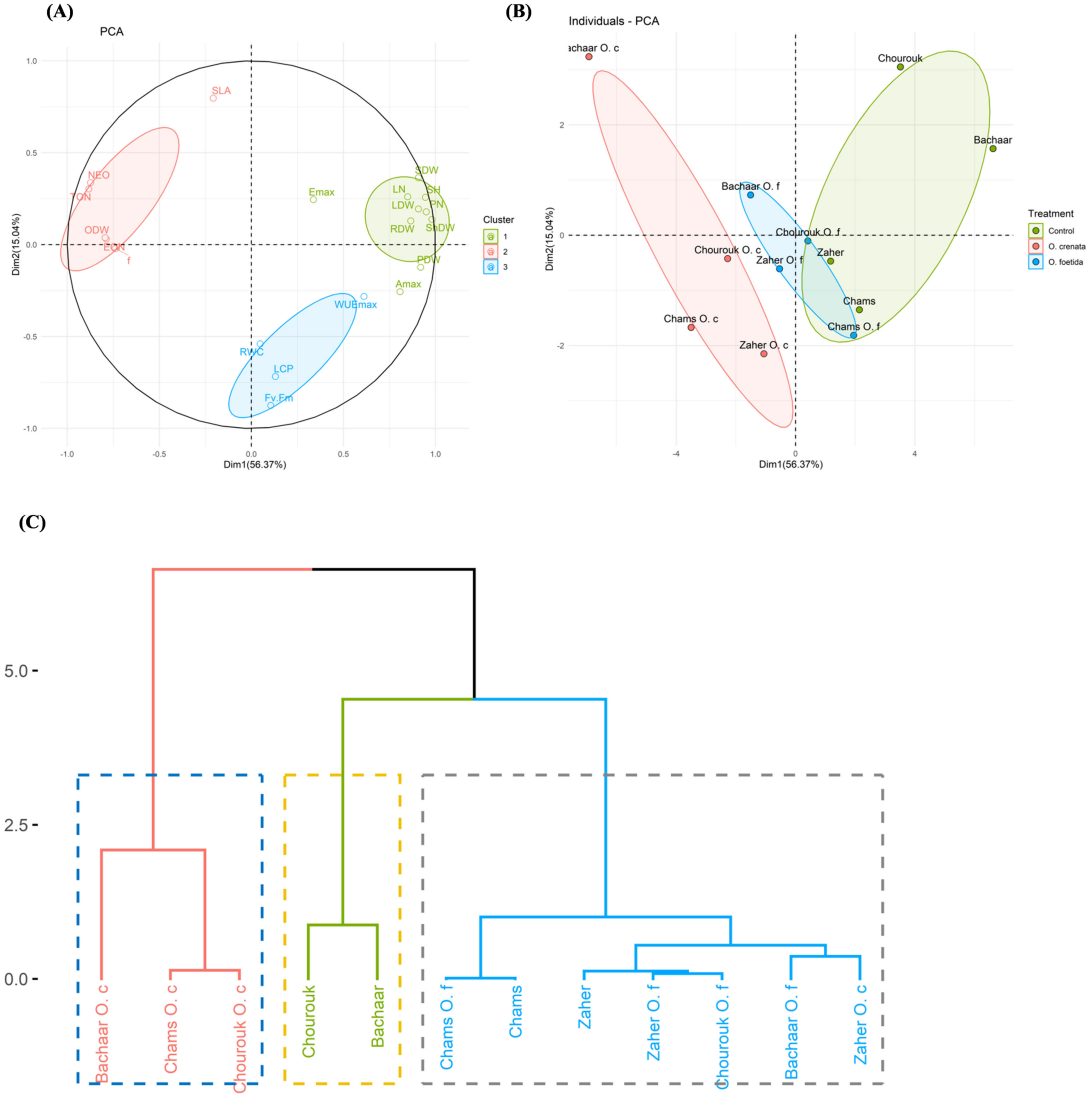
Biplots of the first two dimensions of the principal component analysis (PCA) for the four genotypes based on their yield components and physiological response under three conditions, i.e., control, *O. foetida* infection, and *O. crenata* infection **(A, B)**, and the hierarchical clustering analysis (HCA) **(C)** of these genotypes based on the Euclidean metric calculated using 20 yield components and physiological parameters under the three conditions. For the abbreviations, see **Tables 1**, **3**.

**Figure 5** presents the PCA plot delineating the response of faba bean plants across three conditions: non-infection, *O. foetida* infection, and *O. crenata* infection. The first two principal components, PC1 (56.37%) and PC2 (15.04%), contributed 71.41% of the total variation (**Table 5**). PC1 was positively correlated with yield components, such as ShDW (r=0.98), SH and PN (r=0.95), and LDW and SDW (r=0.91), and photosynthetic traits, including A_max_ (r=0.81) and WUE_max_ (r=0.61). PC1 was negatively correlated with infection parameters including TON (r=-0.88), NEO (r=-0.87), EON, and ODW (r=-0.79). In contrast, PC2 showed a negative correlation with F_v_/F_m_ (r=-0.88) and LCP (r=-0.72), while it showed a positive correlation with SLA (r=0.80). Within the three conditions evaluated, ShDW and F_v_/F_m_ showed the highest contribution to PC1 and PC2 variation, with coefficients of 0.98 and -0.88, respectively. Based on this comprehensive assessment, it is evident that ShDW and F_v_/F_m_ emerge as pivotal discriminative parameters for distinguishing the four faba bean varieties across the control, *O. foetida*, and *O. crenata* conditions.

**Table 5:** Eigenvectors, eigenvalues, and total and cumulative variability of the first three principal components under the control, *O. foetida* and *O. crenata* infection conditions.

	PC1	PC2	PC3
Eigenvalue	11.27	3.01	2.13
Variability (%)	56.37	15.04	10.65
Cumulative (%)	56.37	71.41	82.06
Trait	Eigenvector		
RDW	0.87	0.13	0.34
ShDW	0.98	0.14	0.01
SDW	0.91	0.36	0.14
LDW	0.91	0.19	0.18
PDW	0.92	-0.12	-0.22
LN	0.85	0.26	0.33
PN	0.95	0.18	0.08
SH	0.95	0.26	-0.16
NEO	-0.87	0.34	0.28
EON	-0.79	0.03	0.51
TON	-0.88	0.30	0.32
ODW	-0.79	0.04	0.50
F_v_/F_m_	0.11	-0.88	0.13
A_max_	0.81	-0.26	0.31
Φ	-0.74	-0.02	-0.24
LCP	0.13	-0.72	-0.09
E_max_	0.34	0.24	-0.12
WUE_max_	0.61	-0.28	0.61
SLA	-0.21	0.80	0.29
RWC	0.05	-0.54	0.65

For the abbreviations, see **Tables 1** and **3**.

The checks were positioned on the positive side of PC1 and showed correlations with yield components. Conversely, the *O. crenata* and *O. foetida* treatments were situated on the negative side of PC1 and exhibited correlations with infection parameters, except for the Chams and Chourouk varieties under *O. foetida* infection. **Figure 5C** presents the HCA. This dendrogram grouped the varieties into three clusters: Cluster 1 (Bachaar *O. crenata*, Chams *O. crenata*, and Chourouk *O. crenata*), Cluster 2 (Chourouk and Bachaar) and Cluster 3 (Chams, Chams *O. foetida*, Zaher, Zaher *O. foetida*, Chourouk *O. foetida*, Bachaar *O. foetida*, and Zaher *O. crenata*).

**Figure 6:**
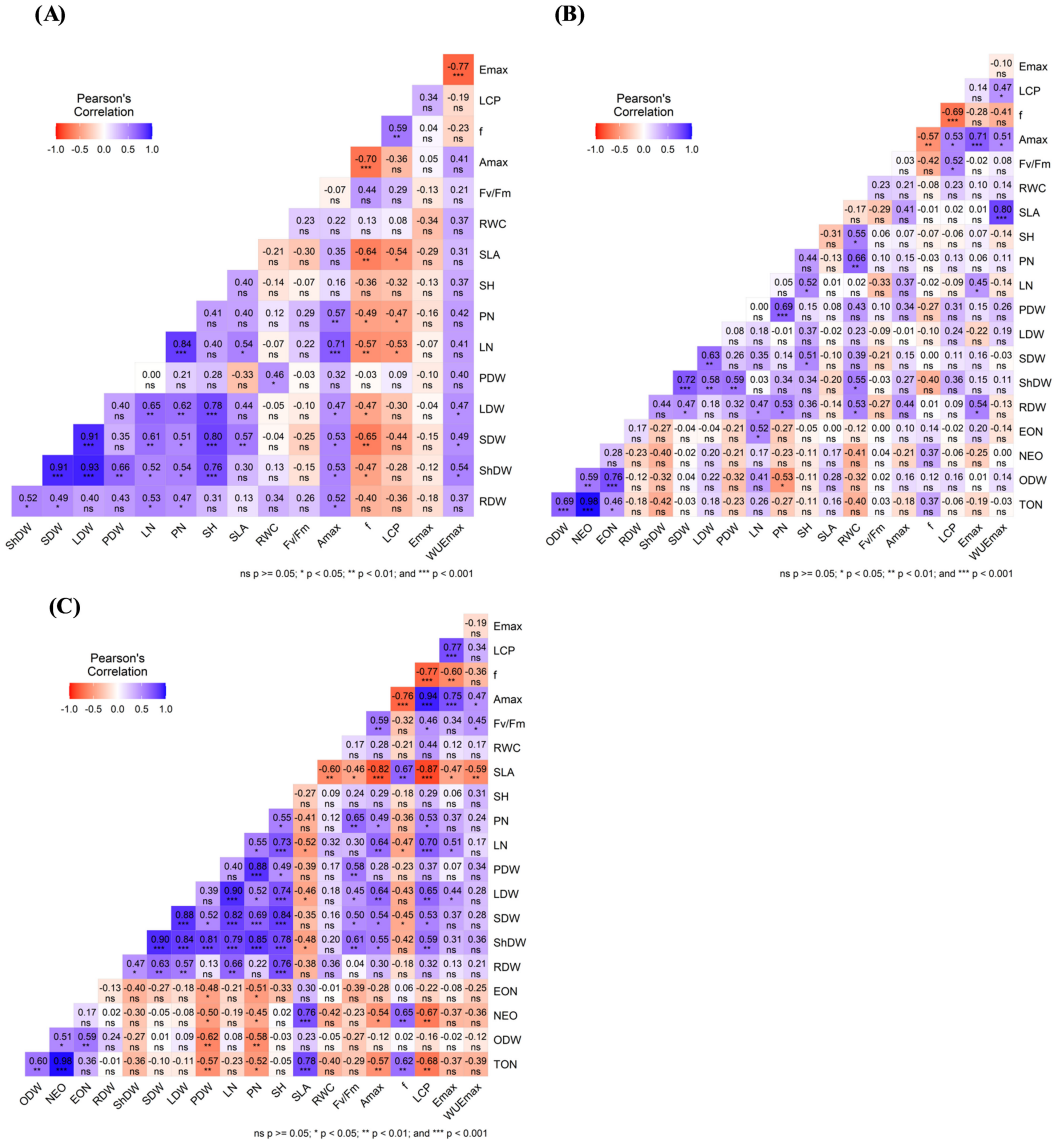
Correlation analysis using Pearson’s correlation coefficient (r) among yield components and physiological parameters under the control **(A)**, *O. foetida* **(B)**, and *O. crenata* **(C)** treatments. ***, **, * indicate correlations significant at the 0.001, 0.01, and 0.05 levels, respectively (Tukey’s test). For the abbreviations, see **Tables 1**, **3**.

**Figure 6** presents Pearson’s correlation coefficients (r) to elucidate the interrelations among various variables. For the non-infection conditions, the correlation matrix demonstrated positive significant correlations between ShDW and several yield components such as LDW (r = 0.93***), SDW (r = 0.91***), SH (r= 0.76***), PDW (r=0.66**), PN (r=0.54*), and LN (r=0.52*). There was also a significant correlation between yield components and physiological parameters, especially A_max_, Φ, and WUE_max_. No significant correlations between F_v_/F_m_ and all the other parameters were observed (**Figure 6A**). Under *O. foetida* infection (**Figure 6B**), notable positive correlations between ShDW and yield components and physiological parameters were only observed for SDW (r=0.72***), LDW (r=0.58**), PDW (r=0.59**), and RWC (r=0.55*). No significant correlations were observed between yield components and infection and physiological parameters. Regarding *O. crenata* infection, ShDW revealed a highly positive correlation with yield components and physiological parameters, such as SDW (r=0.9***), PN (r=0.85***), LDW (r=0.84***), PDW (r=0.81***), LN (r=0.79***), SH (r=0.78***), F_v_/F_m_ (r=0.61**), LCP (r=0.59*), and A_max_ (r=0.55*). We also found significant negative correlations between the infection traits and yield components such as TON with PDW (r=-0.57**) and PN (r=-0.52*), EON with PN (r=-0.51*) and PDW (r=-0.48*), and NEO with PDW (r=-0.50*) and PN (r=-0.45*). Similarly, A_max_ was negatively correlated with infection parameters such as TON (r=-0.57**) and NEO (r=-0.54*). Similarly, physiological parameters, especially Amax and F_v_/F_m_, showed a positive correlation with yield components such as SDW (r=0.5* and r=0.54*) and PN (r=0.65** and r=0.49*) (**Figure 6C**).

## 4 Discussion

Broomrape poses a significant challenge to legume cultivation in the Mediterranean region. In Tunisia, *O. foetida* and *O. crenata* represent a significant hindrance to faba bean cultivation. In previous studies, some newly developed faba bean varieties exhibited tolerance to broomrape infection (Amri et al., 2019). Many tolerance mechanisms have been reported, including those that involve a reduced production of germination stimulants for *Orobanche* seeds (strigolactones) as well as reduced tubercle formation and growth (Abbes et al., 2009b, 2010b, 2011; Trabelsi et al., 2016, 2017). Additionally, other biochemical mechanisms related to a more efficient antioxidant response and reduced lipid peroxidation have been characterized in more recent research (Abbes et al., 2020).

In our study, we assessed the responses of four different faba bean varieties, comprising three tolerant varieties (Chourouk, Chams, and Zaher) and one sensitive variety (Bachaar), to *O. foetida* and *O. crenata* infection. In response to both *Orobanche* species, the tolerant varieties showed low infection levels with lower TON, ODW, ShDW, RDW, and LN values, compared to the sensitive Bachaar variety. The limited number of emerged broomrape shoots observed for Bachaar plants infected with *O. foetida* compared to *O. crenata* can be explained by high competition among the underground tubercles (Trabelsi et al., 2016; Amri et al., 2021). Similar findings have been reported with various legume crops including faba bean (Trabelsi et al., 2016; Amri et al., 2019; Abbes et al., 2020), lentil (En-Nahli et al., 2021; En-nahli et al., 2023), chickpea (Nefzi et al., 2016), and grass pea (Abdallah et al., 2020). Furthermore, Demirbas and Acar (2017) and Ennami et al. (2020) showed a significant decrease in shoot and root DW upon *Orobanche* infection. This reduction in host plant biomass could be attributed to a competition for nutrients, often characterized as a source-sink interaction, between the plant’s biological yield and the development of broomrape tubercles. Consequently, broomrape exerts itself as a strong sink, redirecting the flow of nutrients from its host plant to its benefit (Grenz et al., 2005; Trabelsi et al., 2016). Our findings reveal, furthermore, a positive correlation between TON and ODW (r = 0.6**, 0.69***) and NEO (r =0.98***, 0.98***) and a negative correlation between TON and ShDW (r =-0.36, -0.42), and PN (r = -0.52*, -0.27) under infection with *O. crenata* and *O. foetida*, respectively. In accordance with what was reported by Ter Borg et al. (1994), the tubercle number showed a negative correlation with the biomass of the host plant. A score plot and cluster analysis were used to identify the genotypes exhibiting desirable traits. Our results showed genotypic differences in response to infection by both *Orobanche* species. ShDW and F_v_/F_m_ had the highest contribution to the total variation of PC1 (0.98) and PC2 (-0.88). These results align with prior research carried out by Ben Chikha et al. (2016) and Rajhi et al. (2020), who suggested that parameters related to biomass (total fresh mass) and photosynthesis (net CO_2_ assimilation) can be valuable descriptors for evaluating plant tolerance to stress.

Our findings reveal that the impact on biomass and photosynthetic parameters was less pronounced in the tolerant varieties compared to the sensitive variety. Following *O. crenata* infection, Bachaar stood out as the most sensitive, displaying the lowest values for both yield components and physiological traits while concurrently exhibiting the highest values for ODW, TON, NEO, and EON. Chourouk, Chams, and Zaher clustered together in one group that exhibited a significant negative correlation with principal components PC1 and PC2. This cluster showed a good tolerance to both *Orobanche* species and showed low infection parameters, medium productivity, and good physiological performance under *Orobanche* infection. These tolerant varieties effectively maintained their productivity and sustained elevated levels of photosynthetic activity when subjected to parasitic infection by *O. crenata*.

Under *O. foetida* infection, the four varieties clustered together. Bachaar was positively correlated with infection parameters and negatively correlated to yield components. Zaher was negatively correlated with infection parameters and yield components and positively correlated with photosynthetic traits. Both Chams and Chourouk were negatively correlated with infection parameters and positively with yield components and photosynthetic traits with low attachment numbers, medium yield components, and high physiological parameters.

Under free *Orobanche* infection, both Bachaar and Chourouk exhibited the highest productivity, expressed through high pod numbers, pod dry weight, leaf number and DW, and longest shoot height. Additionally, as highlighted by Amri et al. (2019), Chourouk showed good tolerance to *O. crenata* and *O. foetida* in both controlled and field conditions. A previous investigation indicates that the decrease in plant height and biomass under *Orobanche* infection could be attributed to photosynthesis disruption and/or nutrient imbalance (Mauromicale et al., 2008; Shen et al., 2011; Amri et al., 2021). The reduction of CO_2_ assimilation due to *Orobanche* infection could be associated with a reduction in total chlorophyll content. According to Cameron et al. (2005), the decrease in leaf chlorophyll content could lead to reduced antenna size and light absorption which results in a reduced photosynthetic rate. Previous studies using the same varieties showed that *Orobanche* exhibits its negative effect through the reduction of the chlorophyll content (Abbes et al., 2020; Amri et al., 2021). This aligns with existing studies proposing the hypothesis that disrupted photosynthesis is a result of chlorophyll depletion (Mauromicale et al., 2008; Trabelsi et al., 2016; Nefzi et al., 2016).

*Orobanche* infection significantly affected the photosynthetic system of the sensitive variety, Bachaar, through a significant decrease in the F_v_/F_m_ ratio and plant growth. In contrast, the tolerant varieties, Chams, Chourouk, and Zaher, effectively conserved their photosynthetic activity and successfully managed infection conditions. Several studies have shown that *Orobanche* parasitism leads to a significant reduction in the F_v_/F_m_ ratio in sensitive infected host plants, causing damage to PSII electron transport (Mauromicale et al., 2008; Rousseau et al., 2015; Ennami et al., 2020; Amri et al., 2021). Additionally, Vrbničanin et al. (2013) highlighted a similar impact of the parasitic weed *Cuscuta campestris* on various chlorophyll parameters, such as minimal fluorescence (F_o_), the F_v_/F_m_ ratio, effective fluorescence yield (ΦPSII), and variable fluorescence (F_v_) of giant ragweed (*Ambrosia trifida* L.), showing that these parameters can be used as indicators of the effect of *C. campestris* on host plants. Recently, Amri et al. (2021) demonstrated that tolerant varieties exhibited a comparatively lower impact of *O. foetida* on their chlorophyll content index and quantum yield of photosystem II in contrast with sensitive varieties. Thus, the F_v_/F_m_ ratio has been proposed as a useful and practical screening tool for the early detection of parasitic infection, diagnosis, identification, and selection of highly tolerant varieties (Amri et al., 2019, 2021).

The decreases of A_max_ observed in the tolerant varieties Chams, infected by *O. crenata*, and Zaher, infected by *O. foetida*, did not show any negative impact on biomass and seed production. In contrast, for the sensitive Bachaar variety, both *Orobanche* species significantly reduced plant growth, biomass production, and A_max_. Our results showed, furthermore, that the decrease in A_max_ in the leaves of infected Bachaar plants was not concomitant with a reduction in the Φ. Additionally, a negative correlation was observed between Φ and other photosynthetic parameters (A_max_, E_max_, F_v_/F_m_, and LCP). The low photosynthesis performance in the *Orobanche*-infected plants in comparison to the non-infected ones cannot be explained with Φ.

Except for a slight reduction observed in Chourouk plants infected by *O. foetida*, the SLA remained relatively unchanged in the tolerant faba bean varieties when affected by both *Orobanche* species. Regarding the sensitive Bachaar variety, particularly under *O. crenata* infection, the low photosynthetic capacity of the infected plants was linked to a higher SLA, suggesting that leaf dysfunction due to infection could be potentially related to structural or morphological changes. A similar result was reported on sorghum plants infected with *Striga hermonthica* which significantly affected CO_2_ assimilation through reduced leaf areas and photosynthesis rates (Graves et al., 1989). However, contrary findings from other studies suggest that reduced photosynthetic capacity in stressed plants does not consistently align with a higher SLA (Oguchi et al., 2003; Rzigui et al., 2017; Mahjoubi et al., 2020).

Our results revealed that the reduced photosynthetic capacity observed in the leaves of infected Bachaar plants coincided with lower E values. This decrease in transpiration might be linked to the decreased gs in order to maintain the balance between carbon gain and water loss, as suggested by Pearcy (1990) and Lawson and Blatt (2014). These results are similar to those reported by Shen et al. (2007, 2011), Taylor et al. (1996), and Frost et al. (1997), who indicated that reduced gs and E contribute to stomatal limitations, impeding the diffusion of CO_2_ into photosynthetic tissues and subsequently reducing host plant photosynthesis.

Despite the four studied varieties showing decreases in biomass due to *Orobanche* parasitism, there was no significant effect on the host plant’s RWC. According to Amri et al. (2021), the infected host plants adjusted their production and biomass allocation to sustain their physiological functions, maintaining normal and optimal relative water content despite the constraints imposed by the *Orobanche* infection.

On the other hand, both *Orobanche* species significantly reduced the WUE of the sensitive variety, Bachaar, but no effect was observed in all the tested tolerant varieties. The tolerant variety, Chourouk, exhibited the ability to sustain both photosynthesis and water use efficiency under *Orobanche* infection. Similar findings from Hanba et al. (2002); Bidalia et al. (2017); Mahjoubi et al. (2020), and Rajhi et al. (2020) underscored that augmentation of WUE is a pivotal facet of plant adaptation to diverse environments. However, Mendes et al. (2011) reported that acclimation to different environmental conditions involves behaviors geared toward optimizing WUE rather than only maximizing leaf net carbon gain.

Non-stomatal limitations must be considered important factors that alter photosynthesis. The reduced CO_2_ uptake observed in the Bachaar plants under *Orobanche* spp. infection might also be influenced by non-stomatal factors. This finding aligns with outcomes from a study on *M. micrantha*, where the decline in photosynthesis coincided with a reduction in Rubisco content (Shen et al., 2011). Similar results were observed by other authors (Hudson et al., 1992; Lauerer et al., 1993; Stitt and Schulze, 1994; Furbank et al., 1996).

In conclusion, *O. foetida* and *O. crenata* caused a significant decrease in biomass and strongly disturbed photosynthesis at the leaf level in the sensitive Bachaar variety. This disruption was related to a decrease in E, WUE, A_n_, and photoinhibition of PSII. Non-stomatal limitations cannot be excluded and an exploration of the effects of *Orobanche* infection on the fine structure of leaves and chloroplasts and the functioning of the Calvin cycle is recommended. The three tolerant faba bean varieties were able to maintain their biomass and photosynthesis under *Orobanche* infection. Biomass and photosynthesis parameters can be used to evaluate and discriminate between plants under *Orobanche* infection. However, a better understanding of the other tolerance mechanisms, such as biochemical mechanisms in these tolerant varieties during the broomrape-host interaction, in particular, the role of strigolactones and the lignification process as a means of strengthening the cell wall in the control of attachment (tubercles), is needed.

## Data Availability

The raw data supporting the conclusions of this article will be made available by the authors, without undue reservation.

## References

[B1] Abbes Z. Bouallegue A. Trabelsi I. Trabelsi N. Taamalli A. Amri M.. (2020). Investigation of some biochemical mechanisms involved in the resistance of faba bean (*Vicia faba* L.) varieties to Orobanche spp. Plant Protect. Sci. 56, 317–328. doi: 10.17221/103/2019-PPS

[B2] Abbes Z. Kharrat M. Delavault P. Chaïbi W. Simier P. (2009a). Nitrogen and carbon relationships between the parasitic weed *Orobanche foetida* and susceptible and tolerant faba bean lines. Plant Physiol. Bioch. 47, 153–159. doi: 10.1016/j.plaphy.2008.10.004 19036596

[B3] Abbes Z. Kharrat M. Delavault P. Chaïbi W. Simier P. (2009b). Osmoregulation and nutritional relationships between *Orobanche foetida* and faba bean. Plant Signal. Behav. 4, 336–338. doi: 10.4161/psb.4.4.8192 19794856 PMC2664500

[B4] Abbes Z. Kharrat M. Pouvreau J. B. Delavault P. Chaibi W. Simier P. (2010b). The dynamics of faba bean (*Vicia faba* L.) parasitism by *Orobanche foetida* . Phytopathol. Mediterr. 49, 239–248. Available at: http://www.jstor.org/stable/26458597.

[B5] Abbes Z. Kharrat M. Simier S. Chaïbi W. (2007). Characterization of resistance to crenate broomrape (*Orobanche crenata*) in a new small-seeded line of Tunisian faba beans. Phytoprotection. 88, 83–92. doi: 10.7202/018953ar

[B6] Abbes Z. Sellami F. Amri M. Kharrat. M. (2010a). Effect of sowing date on *Orobanche foetida* infection and seed yield of resistant and susceptible faba bean cultivars. Acta Phytopathol. Entomol. Hung. 45, 267–275. doi: 10.1556/APhyt.45.2010.2.3

[B7] Abbes Z. Sellami F. Amri M. Kharrat. M. (2011). Variation in the resistance of some faba bean genotypes to *Orobanche crenata* . Pak. J. Bot. 43, 2017–2021.

[B8] Abbes Z. Trabelsi I. Kharrat M. Amri M. (2019). Intercropping with fenugreek (*Trigonella foenum-graecum*) enhanced seed yield and reduced *Orobanche foetida* infestation in faba bean (*Vicia faba*). Biol. Agricul. Horticul. 35, 238–247. doi: 10.1080/01448765.2019.1616614

[B9] Abdallah F. Kumar S. Amri A. Mentag R. Kehel Z. Mejri R. K.. (2020). Wild *Lathyrus* species as a great source of resistance for introgression into cultivated grass pea (*Lathyrus sativus* L.) against broomrape weeds (*Orobanche crenata* forsk. and *Orobanche foetida* Poir.). Crop Sci. 61, 263–276. doi: 10.1002/csc2.20399

[B10] Amri M. Abbes Z. Trabelsi I. Ghanem M. E. Mentag R. Kharrat M. (2021). Chlorophyll content and fluorescence as physiological parameters for monitoring *Orobanche foetida* Poir. infection in faba bean. PloS One 16, e0241527. doi: 10.1371/journal.pone.0241527 34032807 PMC8148315

[B11] Amri M. Trabelsi I. Abbes Z. Kharrat M. (2019). Release of a new faba Bean variety "Chourouk" resistant to the parasitic plants *Orobanche foetida* Poir, and *Orobanche crenata* Forsk, in Tunisia. Internat. J. Agricul. Biol. 23, 499–505. doi: 10.17957/IJAB/15.0921

[B12] Barrs H. D. Weatherley P. E. (1962). A re-examination of the relative turgidity technique for estimating water deficit in leaves. Aust. J. Biol. Sci. 15, 413–428. doi: 10.1071/BI9620413

[B13] Ben Chikha M. Hessini K. Nefissi O. R. Ghorbel A. Zoghlami N. (2016). Identification of barley landraces genotypes with contrasting salinity tolerance at vegetative growth stage. Plant Biotechnol-Nar. 33, 287–295. doi: 10.5511/plantbiotechnology.16.0515b PMC6565946 31274990

[B14] Bidalia A. Hanief M. Rao K. S. (2017). Tolerance of *Mitragyna parvifolia* (Roxb.) Korth seedlings to NaCl salinity. Photosynthetica. 55, 231–239. doi: 10.1007/s11099-016-0224-8

[B15] Bouraoui M. Abbes Z. Rouissi M. Abdi N. Hemissi I. Kouki S.. (2016). Effect of rhizobia inoculation, N and P supply on *Orobanche foetida* parasitizing faba bean (*Vicia faba* minor) under field conditions. Biocontrol Sci. Techn. 26, 776–791. doi: 10.1080/09583157.2016.1157137

[B16] Cameron D. D. Hwangbo J. K. Keith A. M. Geniez J. M. Kraushaar D. Rowntree J.. (2005). Interactions between the hemiparasitic angiosperm *Rhinanthus* minor and its hosts: from the cell to the ecosystem. Folia Geobot. 40, 217–229. doi: 10.1007/BF02803236

[B17] Demirbas S. Acar O. (2017). Physiological and biochemical defense reactions of Arabidopsis thaliana to *Phelipanche ramosa* infection and salt stress. Fresenius Environ. Bull. 26, 2275–2268.

[B18] D.G.P.A (2022). Statistiques de la Direction Générale de la Production Agricole (DGPA) (Tunisia: Ministry of Agriculture, water resources and fisheries).

[B19] En-Nahli Y. El Arroussi H. Kumar S. Bouhlal O. Mentag R. Hejjaoui K.. (2021). Resistance to *Orobanche crenata* Forsk. in lentil (*Lens culinaris* Medik.). Exploring some potential altered physiological and biochemical defense mechanisms. J. Plant Interact. 16, 321–331. doi: 10.1080/17429145.2021

[B20] En-nahli Y. Hejjaoui K. Mentag R. Es-safi N. E. Amri M. (2023). Large Field Screening for Resistance to Broomrape (*Orobanche crenata* Forsk.) in a Global Lentil Diversity Panel (GLDP) (*Lens culinaris* Medik.). Plants. 12, 2064. doi: 10.3390/plants12102064 37653981 PMC10222529

[B21] Ennami M. Mansi M. J. Briache F. Z. Oussible N. Gaboun F. Ghaouti L.. (2020). Growth-defense tradeoffs and source-sink relation explain the responses of susceptible and resistant faba bean and lentil genotypes to infection by *Orobanche crenata* . Crop Prot. 127, 104924. doi: 10.1016/j.cropro.2019.104924

[B22] Frost D. L. Gurney A. L. Press M. C. Scholes J. D. (1997). *Striga hermonthica* reduces photosynthesis in sorghum: the importance of stomatal limitations and a potential role for ABA? Plant Cell Environ. 20, 483–492. doi: 10.1046/j.1365-3040.1997.d01-87.x

[B23] Furbank R. T. Chitty J. A. Von Caemmerer S. Jenkins C. (1996). Antisense RNA inhibition of rbcS gene expression reduces Rubisco level and photosynthesis in the C4 plant *Flaveria bidentis* . Plant Physiol. 111, 725–734. doi: 10.1104/pp.111.3.725 12226324 PMC157888

[B24] Graves J. D. Press M. C. Stewart G. R. (1989). A carbon balance model of the sorghum-*Striga hermonthica* host-parasite association. Plant Cell Environ. 12, 101–107. doi: 10.1111/j.1365-3040.1989.tb01921.x

[B25] Grenz J. H. MansChadi A. M. Uygur F. N. Sauerborn J. (2005). Effects of environment and sowing date on the competition between faba bean (*Vicia faba*) and the parasitic weed *Orobanche crenata* . Field Crop Rec. 93, 300–313. doi: 10.1016/j.fcr.2004.11.001

[B26] Hanba Y. T. Kogami H. Terashima I. (2002). The effect of growth irradiance on leaf anatomy and photosynthesis in Acer species differing in light demand. Plant Cell Environ. 25, 1021–1030. doi: 10.1046/j.1365-3040.2002.00881.x

[B27] Hudson G. S. Evans J. R. von Caemmerer S. Arvidsson Y. B. Andrews T. J. (1992). Reduction of ribulose-1,5-bisphosphate carboxylase/oxygenase content by antisense RNA reduces photosynthesis in transgenic tobacco plants. Plant Physiol. 98, 294–302. doi: 10.1104/pp.98.1.294 16668627 PMC1080182

[B28] Kharrat M. Abbes Z. Amri M. (2010). A new faba bean small seeded variety “Najeh” tolerant to Orobanche registered in the Tunisian catalogue. Tun. J. Plant Protect. 5, 125–130.

[B29] Lanquar V. Ramos M. S. Lelièvre F. Barbier-Brygoo H. Krieger-Liszkay A. Krämer U.. (2010). Export of vacuolar manganese by AtNRAMP3 and AtNRAMP4 is required for optimal photosynthesis and growth under manganese deficiency. Plant Physiol. 152, 1986–1999. doi: 10.1104/pp.109.150946 20181755 PMC2850043

[B30] Lauerer M. Saftic D. Quick W. P. Labate C. Fichtner K. Schulze E.. (1993). Decreased ribulose-1,5-bisphosphate carboxylase-oxygenase in transgenic tobacco transformed with “antisense“rbcS. VI. Effect on photosynthesis in plants grown at different irradiance. Planta. 190, 332–345. doi: 10.1007/BF00196962

[B31] Lawson T. Blatt M. R. (2014). Stomatal size, speed, and responsiveness impact on photosynthesis and water use efficiency. Plant Physiol. 164, 1556–1570. doi: 10.1104/pp.114.237107 24578506 PMC3982722

[B32] Mahjoubi Y. Rzigui T. Ben Massoud M. Kharbech O. Loussaief N. Chaoui A.. (2020). Leaf gas exchange of bean (*Phaseolus vulgaris* L.) seedlings subjected to manganese stress. Russ. J. Plant Physiol. 67, 168–174. doi: 10.1134/S1021443720010100

[B33] Mauromicale G. Monaco A. L. Longo A. M. G. (2008). Effect of branched broomrape (*Orobanche ramosa*) infection on the growth and photosynthesis of tomato. Weed Sci. 56, 574–581. doi: 10.1614/WS-07-147.1

[B34] Mendes M. M. Gazarini L. C. Rodrigues M. L. (2011). Acclimation of *Myrtus communis* to contrasting Mediterranean light environments—effects on structure and chemical composition of foliage and plant water relations. Environ. Exp. Bot. 45, 165–178. doi: 10.1016/S0098-8472(01)00073-9 11275224

[B35] Nefzi F. Trabelsi I. Amri M. Triki E. Kharrat M. Abbes Z. (2016). Response of some chickpea (Cicer arietinum L.) genotypes to Orobanche foetida Poir. parasitism. Chil. J. Agric. Res. 76, 170−178. doi: 10.4067/S0718-58392016000200006

[B36] Oguchi R. Hikosaka. K. Hirose T. (2003). Does the photosynthetic light-acclimation need change in leaf anatomy? Plant Cell Environ. 26, 505–512. doi: 10.1046/j.1365-3040.2003.00981.x

[B37] Pearcy R. W. (1990). Sunflecks and photosynthesis in plant canopies. Plant Cell Environ. 41, 421–453. doi: 10.1146/annurev.pp.41.060190.002225

[B38] Rajhi I. Ben Moussa S. Neji I. Baccouri B. Ben Chikha M. Chammakhi. (2020). Photosynthetic and physiological responses of small seeded faba bean genotypes (*Vicia faba* L.) to salinity stress: identification of a contrasting pair towards salinity. Photosynthetica. 58, 174–185. doi: 10.32615/ps.2019.152

[B39] Rousseau C. Hunault G. Gaillard S. Bourbeillon J. Montiel G. Simier P.. (2015). A web resource for theexploration of large chlorophyll fluorescence image datasets. Plant Methods 11, 1–12. doi: 10.1186/s13007-015-0068-4 25866549 PMC4392743

[B40] Rzigui T. Cherif J. Zorrig W. Khaldi A. Nasr Z. (2017). Adjustment of photosynthetic carbon assimilation to higher growth irradiance in three-year-old seedlings of two Tunisian provenances of cork oak (*Quercus suber* L.). Iforest. 10, 618–624. doi: 10.3832/ifor2105-010

[B41] Shen H. Hong L. Chen H. WH Y. Lei Cao H. Wang Z. M. (2011). The response of the invasive weed *Mikania micrantha* to infection density of the obligate parasite *Cuscuta campestris* and its implications for biological control of *M. micrantha* . Bot. Stud. 52, 89–97.

[B42] Shen H. Lan H. Wanhui Y. Honglin C. Zhangming W. (2007). The influence of the holoparasitic plant *Cuscuta campestris* on the growth and photosynthesis of its host *Mikania micrantha* . J. Exp. Bot. 58, 2929–2937. doi: 10.1093/jxb/erm168 17656466

[B43] Shen H. Prider J. Facelli J. Watling J. R. (2010). The influence of the hemiparasitic angiosperm *Cassytha pubescens* on photosynthesis of its host *Cytisus scoparius* . Funct. Plant Biol. 37, 14–21. doi: 10.1071/fp09135

[B44] Stitt M. Schulze D. (1994). Does Rubisco control the rate of photosynthesis and plant growth? An exercise in molecular ecophysiology. Plant Cell Environ. 17, 465–487. doi: 10.1111/j.1365-3040.1994.tb00144.x

[B45] Taylor A. Martin J. Seel W. E. (1996). Physiology of the parasitic association between maize and witchweed (*Striga hermonthica*): is ABA involved? J. Exp. Bot. 47, 1057–1065. doi: 10.1093/jxb/47.8.1057

[B46] Ter Borg S. J. Willemsen A. Khalil S. A. Saber H. A. Verkleij J. A. C. Pieterse A. H. (1994). Field study of the interaction between *Orobanche crenata* Forsk. and some new lines of *Vicia faba* L. in Egypt. Crop Prot 13, 611–616. doi: 10.1016/0261-2194(94)90007-8

[B47] Trabelsi I. Abbes Z. Amri M. Kharrat M. (2015). Performance of faba bean genotypes with *Orobanche foetida* and *Orobanche crenata* infestation in Tunisia. Chil. J. Agr. Res. 75, 27–34. doi: 10.4067/S0718-58392015000100004

[B48] Trabelsi I. Abbes Z. Amri M. Kharrat M. (2016). Study of some resistance mechanisms to Orobanche spp. infestation in faba bean (*Vicia faba* L.) breeding lines in Tunisia. Plant Prod. Sci. 19, 562–573. doi: 10.1080/1343943X.2016.1221734

[B49] Trabelsi I. Yoneyama K. Abbes Z. Xie X. Amri M. Kharrat M. (2017). Characterization of strigolactones produced by *Orobanche foetida* and *Orobanche crenata* resistant and -susceptible faba bean genotypes and effect of phosphorous, nitrogen, and potassium deficiencies on strigolactone production. South Afr. J. Bot. 108, 15–22. doi: 10.1016/j.sajb.2016.09.009

[B50] Triki E. Trabelsi I. Amri M. Nefzi F. Kharrat M. Abbes Z. (2018). Effect of benzothiadiazole and salicylic acid resistance inducers on *Orobanche foetida* infestation in *Vicia faba* . Tun. J. Plant Protect. 13, 113–125.

[B51] Vrbničanin S. P. Sarić-Krsmanović M. M. Božić D. M. (2013). The Effect of Field Dodder (*Cuscuta campestris* Yunck.) on Morphological and Fluorescence Parameters of Giant Ragweed (*Ambrosia trifida* L.). Pesticidi i Fitomedicina. 28, 57–62. doi: 10.2298/PIF1301057V

[B52] Watling J. R. Press M. C. (2000). Infection with the parasitic angiosperm *Striga hermonthica* influences the response of the C3 cereal *Oryza sativa* to elevated CO_2_ . Global Change Biol. 6, 919–930. doi: 10.1046/j.1365-2486.2000.00366

